# Roles of Lipoxygenases in Cardiovascular Diseases

**DOI:** 10.1007/s12265-025-10605-2

**Published:** 2025-03-25

**Authors:** Ting Liu, Ding Ai

**Affiliations:** 1 Key Laboratory of Immune Microenvironment and Disease (Ministry of Education) and Department of Physiology and Pathophysiology, Tianjin Medical University, Tianjin, 300070 China; 2 State Key Laboratory of Experimental Hematology, National Clinical Research Center for Blood Diseases, Key Laboratory of Immune Microenvironment and Disease (Ministry of Education), Tianjin Institute of Cardiology, the Province and Ministry Co-Sponsored Collaborative Innovation Center for Medical Epigenetics, The Second Hospital of Tianjin Medical University, Tianjin Medical University, Tianjin, 300070 China; 3 Department of Cardiology, Tianjin Medical University General Hospital, 154, Anshan Road, Heping District, Tianjin Heping District, Tianjin, 300052 China

**Keywords:** Lipoxygenase, Arachidonic acid, Leukotrienes, Cardiovascular diseases

## Abstract

Lipoxygenases (LOXs) are a family of dioxygenases that catalyze the peroxidation of polyunsaturated fatty acids, such as linoleic acid and arachidonic acid, initiating the synthesis of bioactive lipid mediators. The LOX-mediated production of these bioactive molecules in various cell types plays a critical role in the pathophysiology of cardiovascular diseases, including atherosclerosis, hypertension, and myocardial ischemia–reperfusion injury. In this review, we summarize the roles of LOXs and their products in different cardiovascular cells and conditions, offering valuable insights may contribute to the development of novel therapeutic strategies for cardiovascular diseases.

**Graphical Abstract:**

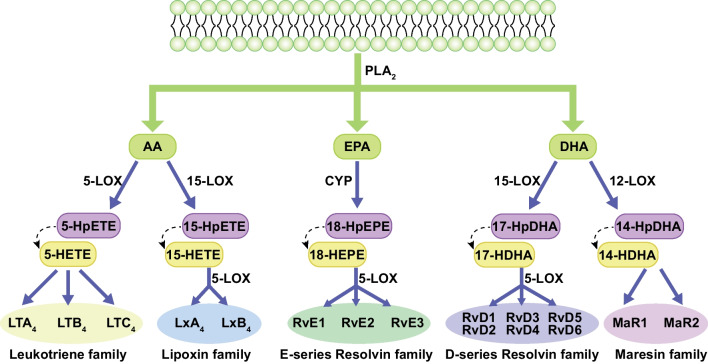

## Introduction

Lipoxygenases (LOXs) are a class of non-heme iron dioxygenases that oxidize polyunsaturated fatty acids (PUFAs) by catalyzing the insertion of molecular oxygen (O_2_) into PUFAs, generating various conjugated hydroperoxides [1]. To date, six functional arachidonate lipoxygenase (*ALOX*) genes encoding six LOX isoforms—5-LOX, 15-LOX-1, 15-LOX-2, 12-LOX, 12R-LOX, and e-LOX-3—have been identified in humans [2, 3]. The enzymatic activity of each *ALOX* isoform is regulated based on its tissue distribution and cell-type-specific expression. *ALOX12B*, *ALOXE3*, and *ALOX15B* are predominantly expressed in epithelial cells, whereas *ALOX5*, *ALOX12*, and *ALOX15* are mainly found in hematopoietic and immune cells [4]. LOX enzymes catalyze the regioselective and stereoselective insertion of O_2_ into PUFA-specific acyl chains, producing highly enriched hydroperoxide derivatives. For instance, arachidonic acid (AA) and linoleic acid (LA) are converted into hydroperoxyeicosatetraenoic acid (HPETEs) and hydroxyoctadecadienoic acids (HODEs), respectively [5]. HPETEs can then be further metabolized into hydroxyeicosatetraenoic acids (HETEs) by glutathione peroxidases or converted into leukotrienes (LTs), lipoxins, and hepoxilins. The formation of LOX-derived metabolites initiates biosynthetic pathways involved in inflammation and immunity. In mammals, LOX products such as LTs and lipoxins play crucial roles in inflammation and innate immune responses [6, 7]. Consequently, they are implicated in the development and progression of various cardiovascular diseases [8, 9]. Table 1 provides an overview of the cellular localization and metabolites of LOXs associated with cardiovascular diseases. A deeper understanding of LOXs enzymes and their metabolites in intracellular signaling pathways relevant to cardiovascular pathology may reveal novel therapeutic strategies for preventing disease progression. In this review, we summarize the pathophysiological roles of LOX family members and their metabolites in different cell types and cardiovascular diseases.

**Table 1:** Cellular localization and products of lipoxygenases associated with cardiovascular diseases

LOXs	Tissue location		Main products	Related cardiovascular diseases
5-LOX		Monocytes, macrophages, neutrophils, mast cells, B-lymphocytes	5(S)-HPETE	Upregulated in atherosclerosis[119, 120] Upregulated in hypertension[110, 121] Increased 5-LOX translocation in myocardial injury [122] Stimulates angiogenesis [123]
15-LOX-1		Macrophages, eosinophils	15(S)-HPETE	Promotes endothelial inflammation [124] Regulates blood pressure [125, 126] Aggravates I/R injury [8, 102] Pro-angiogenic [115, 127]
15-LOX-2		Macrophages	15(S)-HPETE	Pro-atherosclerotic [128]
12-LOX		SMCs, ECs	12(S)-HPETE	Pro-atherosclerotic vascular inflammation [129] Variants (R261Q) in hypertension [130] Regulates hypoxic angiogenesis [60]

Major products are those generated by metabolizing FAs

## Human Lipoxygenases Family and Their Metabolites

### 5-Lipoxygenase

**Fig. 1:**
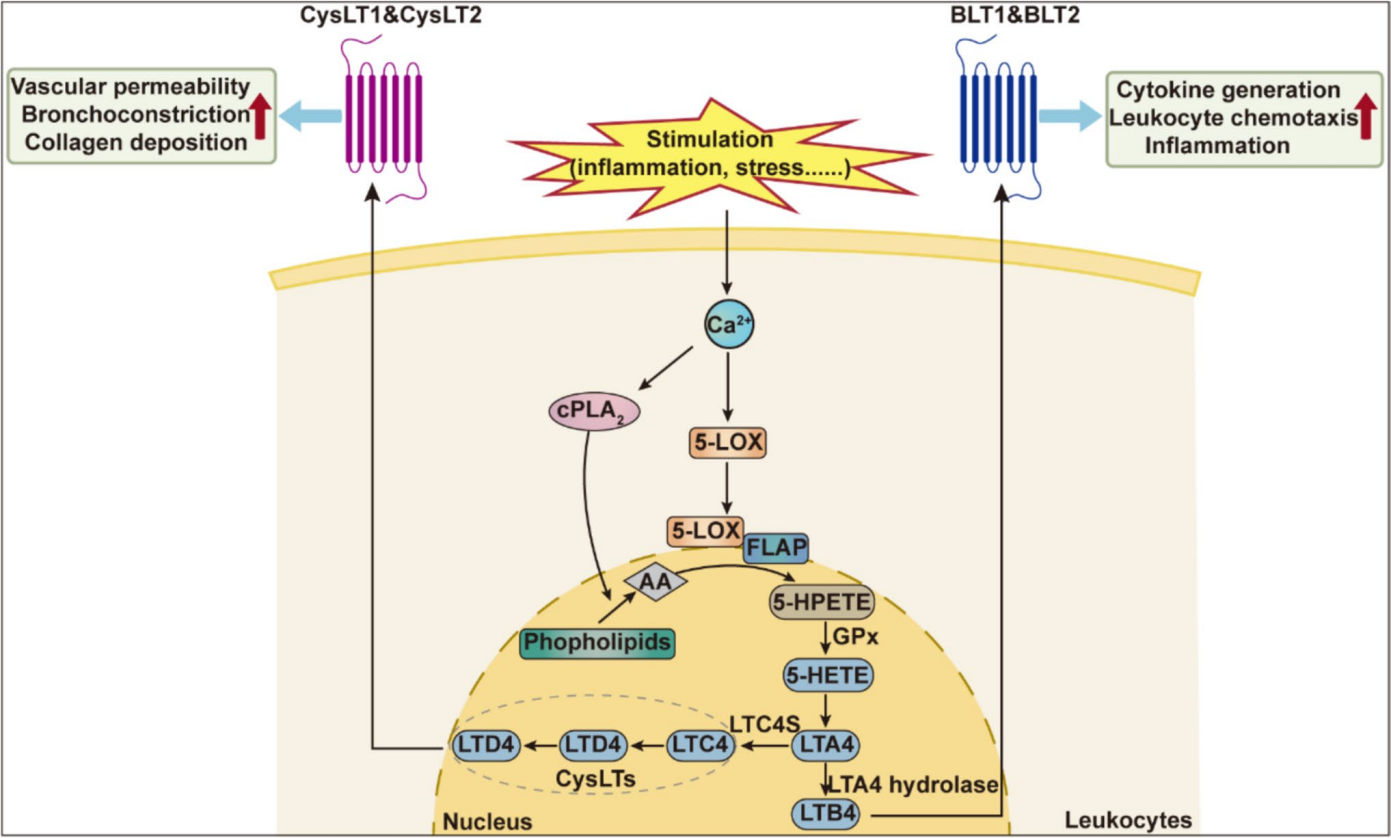
Upon stimulation, increased intracellular Ca^2+^ contributes to the binding of 5-LOX and FLAP, thus further increasing the production of cellular LTs and CysLTs. GPx, Glutathione peroxidase; LTC4S, LTC4 synthase

N-3 and n-6 fatty acids (FAs) and their bioactive metabolites play a crucial role in the inflammatory cascade [10]. The metabolism of FAs into eicosanoids requires the activity of LOXs, cyclooxygenases, or cytochrome P450 (CYP450). AA, a major n-6 FA, is metabolized through the 5-LOX pathway, leading to the synthesis of leukotriene A4 (LTA4) and 5-HETE from 5-HPETE. In contrast, when LA serves as the substrate, LOX enzymes catalyze its conversion into 9-HODE, a compound known to modulate platelet activation [11]. Upon cell stimulation, an increase in intracellular calcium triggers the translocation of cytoplasmic phospholipase A_2_ (cPLA_2_) and 5-LOX from the cytoplasm to the nuclear envelope. At the nuclear envelope, 5-LOX binds to 5-LOX activating protein (FLAP), a transmembrane protein with three structural domains essential for leukotriene biosynthesis. In leukocytes, cPLA_2_ releases AA from membrane phospholipids, and FLAP transfers the liberated AA to 5-LOX, which then catalyzes its sequential oxidation into 5-HPETE [12–14]. LTA4 can be further metabolized into either leukotriene B4 (LTB4) by LTA4 hydrolase or leukotriene C4 (LTC4) by LTC4 synthase. LTC4 are subsequently converted into leukotriene D4 (LTD4) and leukotriene E4 (LTE4) in sequential steps. These three metabolites—LTC4, LTD4 and LTE4—are collectively referred to as cysteinyl leukotrienes (CysLTs) due to their cysteine moiety [15]. Leukotrienes are signaling molecules involved in vascular homeostasis via G protein-coupled receptors, including the LTB4 receptors (BLT1 and BLT2) and the CysLT receptors (CysLT1 and CysLT2))) [16]. The enzyme pathway through which 5-LOX catalyzes the conversion of AA into its metabolites is illustrated in Fig. 1.

Eicosapentaenoic acid (EPA) and docosahexaenoic acid (DHA), as key n-3 FAs, serve as primary substrates for the biosynthesis of bioactive compounds. Through the 5-LOX pathway, EPA is metabolized into 5-hydroxyeicosapentaenoic acid (5-HEPE) and E-series resolvins (RvEs), while DHA is converted into 4-hydroxydosahexaenoic acid (4-HDHA), 7-hydroxydosahexaenoic acid (7-HDHA) and D-series resolvins (RvDs) [10].

Specialized pro-resolving mediators (SPMs), including lipoxins (LXA4 and LXB4), resolvins (RvDs and RvEs), protectins and maresins, play a critical role in resolving inflammation [17]. LXA4 and LXB4 are synthesized through the dual oxygenation of AA by 15-LOX and 5-LOX. SPMs act as essential regulators that initiate anti-inflammatory processes and promote the resolution of inflammation [18]. Recent studies suggest that many chronic inflammatory conditions arise from incomplete resolution of inflammation [19]. Consequently, enhancing the resolution pathway through SPMs may provide therapeutic benefits for various diseases, particularly inflammation-related cardiovascular conditions.

Emerging evidence indicates that exercise and diet significantly influence FA metabolism and inflammatory status in non-communicable diseases, such as obesity. Exercise has been shown to modulate cardiac FA and SPM levels, while n-3 FA supplementation has demonstrated positive effects on heart health. These findings highlight the importance of physical activity, along with the quantity and quality of dietary FAs, in managing obesity-related diseases [20]. Additionally, sleep has been identified as a key modulator of SPM levels in the infarcted heart post-MI. Studies indicate that sleep disruption elevates inflammatory mediators, such as prostaglandins (PGs), while reducing SPM levels in the infarcted heart. Therefore, adequate sleep is essential for maintaining cardiovascular health and supporting the resolution of inflammation [21].

### 15-Lipoxygenase-1 and 15-Lipoxygenase-2

Members of the 15-LOX family oxidize PUFAs to generate their corresponding hydroperoxyl derivatives [22]. In humans, the two known 15-LOX isoforms are 15-LOX-1 and 15-LOX-2, encoded by the *ALOX15* and *ALOX15B* genes, respectively [23]. Notably, the orthologous *ALOX15* gene in mice encodes a distinct “leukocyte-type” 12-LOX (commonly referred to as 12/15-LOX), which catalyzes the production of 12-HETE and 15-HETE in a 6:1 ratio [24].

15-LOX isoforms are highly expressed in reticulocytes, macrophages, eosinophils, neutrophils, respiratory epithelial cells, and vascular cells [25]. Despite their homology, the functional specificity of these enzymes varies across species. For instance, human 15-LOX-2 exclusively oxygenates AA at the C-15 position, whereas its murine ortholog, also known as 8-LOX, predominantly exhibits 8S-lipoxygenating activity [26]. Human 15-LOX-1 primarily generates 15-HETE, with minor production of 12-HETE, whereas the murine 12/15-LOX predominantly produces 12-HETE, with only small amounts of 15-HETE [22].

15-HETE can be further metabolized by 5-LOX to produce LXA4 and LXB4, both of which possess potent anti-inflammatory and pro-resolving properties [27]. Additionally, the EPA metabolite 18-HEPE can be converted into RvE3, while DHA is metabolized by 15-LOX to generate protectin D1. The synthesis of these specialized pro-resolving lipid mediators highlights the anti-inflammatory role of 15-LOX, which may counteract atherogenesis.

However, 15-LOX has also been implicated in pro-atherogenic processes by promoting the oxidation of low-density lipoprotein (LDL). The expression of 15-LOX-1 is strictly dependent on stimulation by T-helper 2 (Th2) cytokines, particularly interleukin (IL)−4 and IL-13. In contrast, 15-LOX-2 is constitutively expressed in human monocyte-derived macrophages.

### 12-Lipoxygenase and 12R-lipoxygenase

12-LOX, also known as platelet-type 12S-LOX, is predominantly expressed in human platelets and leukocytes, as well as in mouse platelets, megakaryocytes, and skin. This enzyme primarily converts AA into 12S-hydroperoxydicosatetraenoic acid (12(S)-HPETE), which is subsequently reduced to 12(S)-HETE by peroxidases [28]. 12-HETE serves as an important signaling molecule involved in inflammation, immune cell recruitment, and vasoconstriction [29]. Additionally, 12-LOX metabolizes DHA to produce maresin 1, a specialized pro-resolving mediator [30]. 12-LOX has been implicated in the regulation of inflammatory and apoptotic processes, as well as in liver injury, through its mediation of the nuclear factor-kappa B (NF-κB) signaling pathway and caspase-3 activation [31]. Collectively, these findings suggest that 12-LOX plays a significant role in the pathogenesis of various cardiovascular diseases.

The physiological role of 12R-LOX is highly specific due to its restricted expression in epithelial cells. Autosomal recessive congenital ichthyosis, a condition characterized by excessive water loss from the skin epithelium leading to severe dryness, has been linked to mutations in genes encoding 12R-LOX and epidermal lipoxygenase 3 (eLOX-3) [32, 33]. Recent genetic studies have demonstrated that 12R-LOX and eLOX-3 function within the same metabolic pathway, facilitating the metabolism of AA to 12R-HPETE, which is further converted into hepoxilin- and trioxilin-like metabolites essential for keratinocyte differentiation [32]. Thus, the 12R-LOX and eLOX-3 pathways play a crucial role in maintaining epidermal barrier function and regulating lipid metabolism.

### eLipoxygenase-3

Unlike other LOXs, eLOX-3 lacks dioxygenase activity. Instead, its preferred substrate is 12R-HPETE, the primary AA derivative generated by 12R-LOX, from which it produces epoxyeicosatrienoic acid (EET) [34]. Epidermal 12R-LOX and eLOX-3 possess distinct structural and enzymatic characteristics that differentiate them from other LOXs [35]. As previously mentioned, the 12R-LOX–eLOX-3 pathway plays a critical role in maintaining epidermal barrier function [36].

### Distribution and Action of Lipoxygenases in Cardiovascular Cells

#### Lipoxygenases in Cardiomyocytes

In pathological conditions such as ischemia or heart failure, eicosanoids and other bioactive lipid mediators induce maladaptive changes, including inflammation and the activation of multiple gene transcription pathways [37]. These alterations contribute to disease progression. Myocardial membrane phospholipids serve as the primary source of AA, which is released through the hydrolytic activity of cardiac cPLA_2_ [38]. In the heart, AA metabolism occurs predominantly in three cell types—cardiomyocytes, ECs, and VSMCs—which rely on complex intercellular communication and paracrine signaling to regulate blood flow and hemodynamic function [39].

Studies have shown that after myocardial ischemia/infarction, activated LOX enzymes and their metabolites contribute to pathological changes such as cardiac hypertrophy, cardiomyocyte apoptosis, and fibrosis [40]. Following hypoxic stimulation, the intracellular accumulation of reactive oxygen species leads to upregulated 5-LOX expression and activity in H9c2 cardiomyocytes, which in turn increases leukotrienes production and induces cell proliferation [41]. The use of 5-LOX inhibitors has been shown to protect against ischemia-induced H9c2 cardiomyocyte injury by reducing oxidative stress [39].

The LOX-derived metabolite HETEs play a key role in actin cytoskeleton organization by selectively binding to actin fibers, promoting their phosphorylation and polymerization [42]. Additionally, LOX-derived eicosanoids have been reported to directly interact with actin filaments [43]. One study showed that 12-LOX and 12-HETE in cardiomyocytes help maintain an intact actin network, facilitating the translocation of the glucose transporter type 4 (GLUT-4) to the plasma membrane [44]. Furthermore, this interaction has been shown to mitigate high glucose-induced insulin resistance in diabetic hearts. The inhibition of the LOX pathway in adult rat cardiomyocytes abolished insulin-stimulated glucose transport [45]. However, the addition of 12-HETE to LOX-inhibited cardiomyocytes restored insulin responsiveness. The inhibition of 12-LOX impairs the GLUT-4 translocation due to two key factors: first, LOX-catalyzed eicosanoids production enhances actin fiber polymerization, maintaining cytoskeletal integrity; and second, an intact cytoskeleton is required for insulin-dependent GLUT-4 translocation to the plasma membrane [46].

In addition to its enzymatic activity, 12-LOX plays a non-enzymatic regulatory role in cell signaling. In myocardial I/R injury, 12-LOX exacerbates cardiomyocyte damage by increasing 12-HETE production in an enzyme-dependent manner while simultaneously inhibiting AMP-activated protein kinase (AMPK) activity via an enzyme-independent mechanism [47]. The pharmacological inhibition of 12-LOX has been shown to effectively prevent cardiac injury and improve heart function. These findings provide the first evidence suggesting that 12-LOX has an enzyme-independent role in cell signaling during I/R injury, indicating its broader functional significance beyond its catalytic activity.

Our previous studies have also identified a role for myocardial 15-LOX in cardiac I/R injury. Following cardiac injury, 15-LOX expression is significantly upregulated in cardiomyocytes. Increased levels of the 15-LOX-generated metabolite 15-HPETE facilitate the binding of peroxisome proliferator-activated receptor-gamma coactivator-1 alpha (PGC1α) to ring finger protein 34 (RNF34), an E3 ubiquitin ligase, leading to PGC1α degradation. This process results in mitochondrial dysfunction and structural abnormalities, ultimately triggering ferroptosis in cardiomyocytes [8].

#### Lipoxygenases in Endothelial Cells

Human ECs lack the ability to metabolize AA into 5-HETE and LTs due to the absence of 5-LOX expression [48]. However, ECs still actively participate in the metabolism of PUFAs. On one hand, ECs express several receptors, including BLT1, BLT2, CysLT1, and CysLT2 [48]. Upon exposure to proinflammatory cytokines such as IL-1β, and LTB4, the expression of BLT1 and BLT2 on the EC surface is upregulated, promoting a proinflammatory phenotype characterized by the release of monocyte chemotactic protein-1 (MCP-1) [49]. LTB4 receptor antagonists have been shown to partially inhibit LTB4-induced signaling in ECs. On the other hand, ECs possess LTA4 hydrolase activity, allowing them to convert exogenous LTA4—originating from other cellular sources—into.

LTB4 and CysLTs [50]. This process, known as transcellular biosynthesis, has been demonstrated using S35-cysteine-labeled ECs co-cultured with polymorphonuclear leukocytes (PMNL), providing direct evidence of PMNL-derived LTA4 being metabolized into to CysLTs. In these co-cultures, PMNL exhibited significantly higher LTB4 levels compared to PMNL cultured alone, suggesting that interactions between vascular ECs and activated leukocytes influence both the quantity and type of LT synthesized [51].

LTB4 is a potent stimulator of neutrophil adhesion and migration, as well as a functional and chemotactic activator of other immune subtype [52]. In several in vivo and in vitro studies, endothelial stimulation by LTB4 in the presence of neutrophils has been shown to increase vascular permeability. This effect is mediated by heparin-binding proteins released from the LTB4-stimulated neutrophils [53, 54]. Additionally, LTB4 promotes EC migration, tube formation, and vascular endothelial growth factor (VEGF)-induced angiogenesis via BLT2 signaling [55]. The interactions between ECs and neutrophils, as well as endothelial LTB4 synthesis via transcellular intermediate-sharing mechanism, contribute to autocrine activation and paracrine stimulation [56]. Moreover, CysLT synthesis in ECs has been linked to increased expression of endothelial adhesion molecules and the activation of a proinflammatory phenotype [57, 58]. CysLTs also regulate inflammatory and proliferative signaling through CysLT1 and CysLT2 receptors [59].

Although 5-LOX expression is minimal in ECs, the presence of 12-LOX and its metabolite, 12-HETE, has been documented in these cells. In pulmonary arterial endothelial cells, 12-LOX activity increases in response to hypoxic stimulation, leading to elevated 12-HETE production [60]. The accumulation of 12-HETE disrupts endothelial homeostasis, resulting in excessive migration, abnormal tube formation, and impaired apoptosis under hypoxic conditions. Furthermore, 12-HETE has been shown to enhance the expression of CS-1 fibronectin on ECs, which may serve as a key mechanism underlying monocyte adhesion and subsequent inflammation.

#### Lipoxygenases in Vascular Smooth Muscle Cells

Similar to ECs, VSMCs exhibit low 5-LOX activity. However, they are capable of generating LTB4 and LTC4 from LTA4 due to the presence of LTA4 hydrolase and LTC4 synthase, which function downstream in the leukotriene biosynthesis pathway [61]. LTA4 hydrolase possesses two distinct enzymatic activities: it acts as an epoxide hydrolase, using LTA4 as a substrate, and as an aminopeptidase with a shared active center. LTC4 synthase, along with FLAP, belongs to the family of membrane-associated proteins involved in eicosanoid metabolism. This enzyme plays a key role in CysLT synthesis by catalyzing the conjugation of LTA4 with glutathione to form LTC4 [62]. Subsequent enzymatic cleavage of LTC4 results in the formation of LTD4 and LTE4.

Several LOX-derived metabolites have been shown to promote VSMC migration, hypertrophy and fibronectin synthesis [63]. Specific LOX inhibitors have been found to reduce the increase in total protein levels induced by angiotensin II (Ang II) in porcine VSMCs [64]. Furthermore, treatment with 12-HETE leads to an increase in total cellular protein content and fibronectin levels comparable to that induced by Ang II. LOX-derived products also exert pro-inflammatory effects in VSMCs. The linoleic acid metabolite, 13-HPODE, generated by 15-LOX, has been observed to significantly enhance the activation of the redox-sensitive NF-κB in VSMCs. This activation is associated with increased transcription of the genes encoding vascular cell adhesion molecule 1 (VCAM-1), a key inflammatory mediator, and MCP-1, a potent chemokine, via through NF-κB-dependent mechanism [65].

#### Lipoxygenases in Immune Cells

5-LOX plays a critical role in the initial step of LT synthesis and the conversion of AA into the unstable intermediate LTA4 [66]. The enzyme is primarily expressed in myeloid-derived immune cells, including neutrophils, eosinophils, macrophages, and mast cells [67]. Upon stimulation, 5-LOX catalyzes the synthesis of LTB4, a potent inflammatory mediator that recruits and activates leukocytes [48]. Recent research has focused on elucidating the mechanism by which LTB4 functions as a chemoattractant. LTB4 enhances neutrophil activation and directs them to form neutrophil extracellular traps (NETs), a specialized form of cell death known as NETosis. This suggests that 5-LOX may regulate neutrophil death by influencing pathways leading to necrosis and apoptosis [68].

The chemotactic effects of LTB4 are mediated by its receptor BLT1, which is highly expressed on CD4 + and CD8 + T cells [69]. BLT1 facilitates T-cell adhesion to epithelial cells and plays a key role in T-cell recruitment and migration, particularly in conditions such as asthma [70]. In addition to BLT1, peripheral blood T cells also express CysLT1 and CysLT2, which mediate responses to CysLTs. Notably, LTE4 has been shown to activates Th2 cells, amplifying the production of pro-inflammatory cytokines in response to PGD2 [71, 72].

Atherosclerosis is a chronic inflammatory disease characterized by the adhesion and infiltration of various immune cells, including macrophages and T cells [73]. A key event in atherosclerosis pathogenesis is the recognition and uptake of oxidized low-density lipoproteins (oxLDLs) by macrophages, leading to foam cell formation. Studies suggest that 15-LOX contributes to this process by directly oxidizing LDL particles, thereby promoting oxLDL accumulation and accelerating foam cell formation [74].

### Roles of Lipoxygenases in Cardiovascular Diseases

#### Lipoxygenases and Atherosclerosis

Atherosclerosis is a complex, multifactorial disease driven by chronic inflammatory processes that influence its initiation, plaque progression, and eventual rupture [75]. The clinical outcome of inflammation is determined by the balance between pro-inflammatory and pro-resolving lipid mediators [76]. The acute inflammatory response consists of two phases: initiation and resolution [77].

During the initiation phase, chemical messengers such as cytokines, chemokines, and LOX-catalyzed pro-inflammatory lipid mediators, including LTs, promote leukocyte activation and chemotaxis [78]. Targeting 5-LOX and its activating protein, FLAP, has been explored as potential anti-atherosclerotic strategy to reduce plaque inflammation. First, phase II clinical trials have demonstrated that the 5-LOX inhibitor VIA-2291 reduces LT production and plaque area in patients with coronary artery disease [79]. Second, in a randomized controlled trial, individual carrying FLAP or LTA4 hydrolase haplotypes associated with an increased risk of myocardial infarction shown reduced C-reactive protein levels when treated with the FLAP antagonist DG-031 [80].

During the resolution phase, SPMs derived from n-3 FAs (EPA and DHA) or AA exhibit anti-inflammatory and pro-resolving effects by limiting PMNL infiltration and promoting macrophage-mediated clearance of apoptotic PMNL [78]. Clinical evidence suggests that dietary supplementation with n-3 FAs has anti-inflammatory effects and is associated with cardioprotection [81]. As the inflammatory response transitions from initiation to resolution, lipid mediator production shifts from early pro-inflammatory PGs and LTs to SPMs such as lipoxins and resolvins [82]. The balance between AA-derived pro-inflammatory and pro-resolving mediators depends on the subcellular localization of 5-LOX: nuclear 5-LOX, in proximity to LTA4 hydrolase, facilitates the biosynthesis of pro-inflammatory LTB4, whereas cytoplasmic 5-LOX promotes the production of pro-resolving LXA4 [83]. Successful inflammation resolution contributes to pathogens clearance and subsequent tissue repair. However, if resolution is impaired, inflammation may become chronic and persistent [17]. Advanced atherosclerosis is characterized by an imbalance between pro-inflammatory and pro-resolving mechanisms, leading to sustained inflammation and tissue injury [76].

**Fig. 2:**
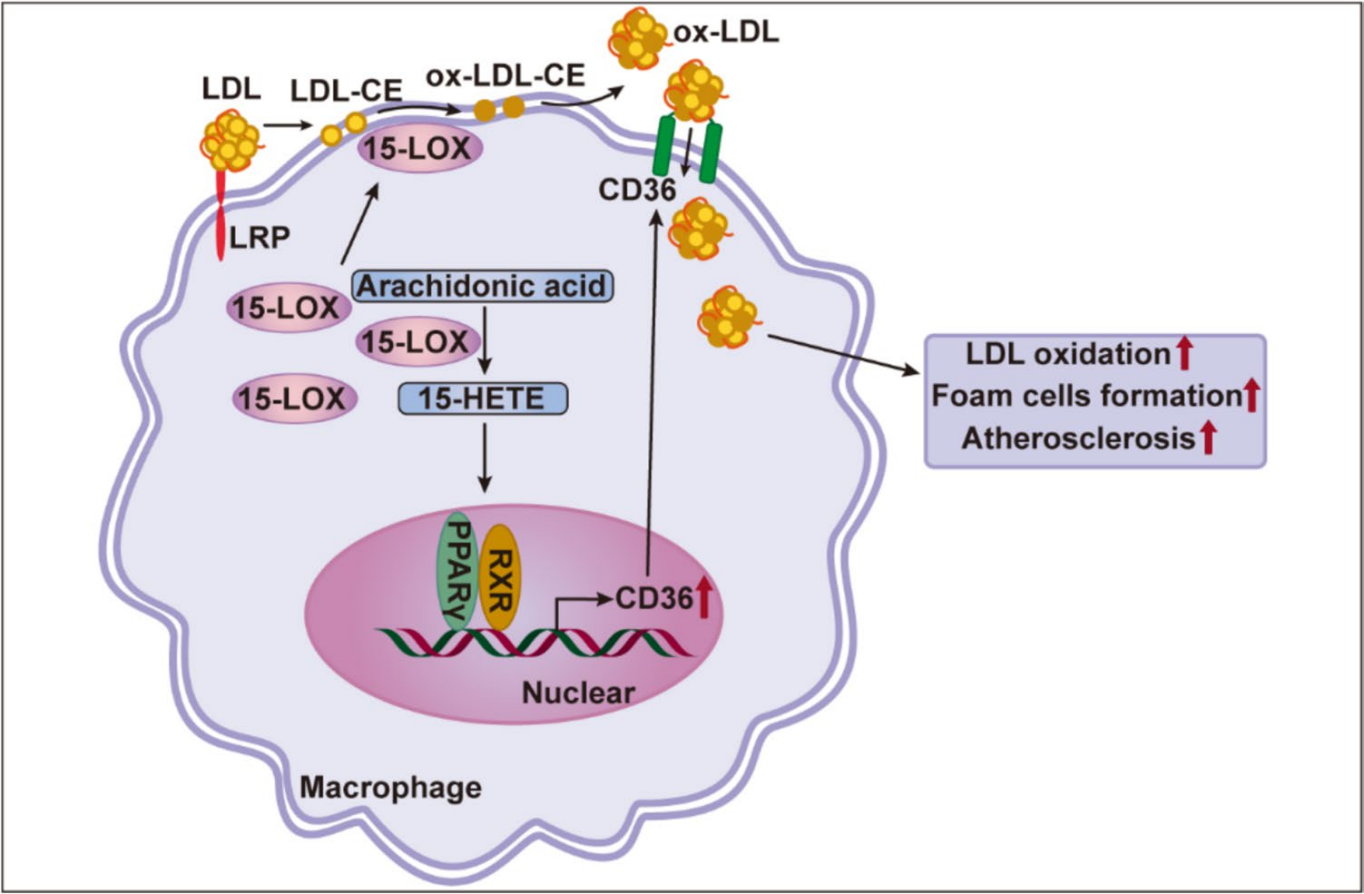
Regulation of LDL oxidation and scavenger receptor CD36 expression by 15-LOX in macrophages. LRP, LDL receptor-related protein; PPARγ, Peroxisome proliferator-activated receptor gamma

The role of 15-LOX in atherosclerosis is twofold, exhibiting pro-inflammatory and anti-inflammatory effects [84]. 15-LOX metabolizes AA into 15-HETE, which can be further processed by 5-LOX to produce pro-resolving mediators LXA4 and LXB4 [27]. Additionally, 15-LOX converts DHA and EPA into protectin D1 and RvE3, respectively, both of which have potent anti-inflammatory effects. However, 15-LOX has also been implicated in pro-atherogenic processes, particularly through its role in LDL oxidation [85]. Studies have shown purified 15-LOX oxidizes cholesteryl linoleate within LDL particles when incubated with LDL. Furthermore, IL-4 and IL-3 have been found to regulate monocyte-mediated LDL oxidation via 15-LOX activation [86]. The metabolite 15-HETE has been demonstrated to upregulate CD36 expression via Peroxisome proliferator-activated receptor gamma (PPARγ), while treatment with the 15-LOX inhibitor PD146176 prevents IL-4-induced CD36 activation [87–89]. Moreover, macrophages exposure to LDL promotes the translocation of 15-LOX to the plasma membrane, where it facilitates LDL oxidation [90]. LDL receptor-related protein (LRP) is essential for this process, as its binding to LDL triggers the translocation of 15-LOX from the cytoplasm to the membrane and facilitates the transfer of linoleic acid esters from LDL particles to the plasma membrane [91]. Once at the membrane, 15-LOX oxidizes linoleic acid esters, and the resulting oxidized cholesterol esters are transported back into LDL particles, initiating a free-radical chain reaction that generates oxLDL. OxLDL is subsequently recognized by scavenger receptors, further promoting foam cell formation and atherogenesis [91]. These findings suggest that macrophage-derived 15-LOX contributes to LDL oxidation and exacerbates atherosclerosis progression by generating reactive hydroperoxides within LDL particles. The effects of 15-LOX in the early stages of atherogenesis are illustrated in Fig. 2. Given the conflicting roles of 15-LOX in atherosclerosis, further investigation is necessary to clarify its impact on human cardiovascular risk assessment.

#### Lipoxygenases and Myocardial Infarction

Inflammation plays a crucial role in both acute myocardial infarction (MI) and chronic heart failure. A timely acute inflammatory response following myocardial injury is essential for clearing damaged and necrotic cardiomyocytes. Our previous findings indicate significantly elevated levels of LTB4 in the plasma of patients with acute MI [92]. LTB4 facilitates PMNL and monocyte adhesion to the vascular endothelium and activates ECs, leading to increased calcium influx and reactive oxygen species production [93]. By disrupting vascular ECs and increases vascular permeability, LTB4 exacerbates vascular injury through these mechanisms. We propose that the increased secretion of LTB4 is mediated by enhanced binding of 5-LOX to FLAP in macrophages during myocardial ischemia. In addition to LTB4, CysLTs have been shown to promote leukocyte adhesion to ECs, VSMC proliferation, and the release of pro-inflammatory cytokines in vitro [94, 95]. Pharmacological interventions targeting these pathways have demonstrated cardioprotective effects. For example, treatment with LY171883, an LTD4/LTE4 receptor antagonist, reversed the ischemia-induced increase in coronary vascular resistance and improved cardiac recovery [96]. Similarly, the CysLT antagonist ONO-1078 and the 5-LOX inhibitor AA-861 significantly reduced infarct size and PMNL infiltration in models of myocardial I/R injury in dogs and rats [97]. However, LOXs also play a reparative role by enhancing SPM synthesis after myocardial injury [98]. While n-6 FAs exacerbate inflammation by promoting pro-inflammatory pathways, n-3 FAs facilitate inflammation resolution and tissue repair within the splenocardiac and cardiorenal networks post-MI. Notably, mice fed a diet enriched with n-3 FAs exhibited elevated SPM levels in the spleen, whereas those fed an n-6 FA-enriched diet had increased levels of pro-inflammatory mediators such as LTB4 [99]. Furthermore, LOXs are essential for SPMs biosynthesis, which plays a key role in activating cardiac repair mechanism and promoting inflammation resolution following MI [100]. Studies demonstrate that administration of RvD1 exerts cardioprotective effects by reducing infarct size and mitigating inflammation post-MI [101].

Although revascularization is the standard treatment for acute MI, the one-year post-MI mortality rate remains at 7%, largely due to additional cardiomyocyte death and structural damage caused by I/R injury. Our previous research has shown that myocardial expression of 15-LOX increases following cardiac I/R injury, leading to the production of 15-HPETE, which induces cardiomyocyte ferroptosis, thereby exacerbating I/R injury [8]. Importantly, these deleterious effects were mitigated by ML351, a selective 15-LOX inhibitor. Additionally, one study demonstrated that deletion of 15-LOX post-MI not only reduced the production of the pro-inflammatory metabolites 12(S)- and 15(S)-HETE but also shifted AA metabolism toward the CYP450 pathway, resulting in increased production of EETs, which possess anti-inflammatory properties [102].

#### Lipoxygenases and Hypertension

Several studies suggest that 15-LOX plays role in regulating vascular tone and may contribute to blood pressure regulation and hypertension [103, 104]. One study demonstrated that AA induces endothelium-dependent relaxation in the rabbit aorta [105]. Since this relaxation is inhibited by nordihydroguaiaretic acid (NDGA), a non-specific LOX inhibitor, it has been proposed that LOX-generated metabolites act as molecular inducers of vasodilation. Research indicates that 15-LOX regulates vascular tone and remodeling by acting on both ECs and VSMCs [103]. Ang II, a known contributor to hypertension, stimulates the 15-LOX-catalyzed production of HETEs in the arteries and kidneys. It has been speculated that these metabolites mediate the involvement of 15-LOX in vascular tone regulation [64, 106]. The 15-LOX-derived metabolite 15(S)-HPETE inhibits prostacyclin synthase activity, leading to reduced prostacyclin levels [64]. Additionally, 15-LOX decreases nitric oxide bioavailability, contributing to vasoconstriction [107]. Conversely, another study found that treating pre-constricted rabbit arteries with AA resulted in vasorelaxation, primarily mediated by 15-LOX-generated metabolites [108]. Furthermore, the lipoxins, which are also generated by 15-LOX, have been reported to promote vasorelaxation [109]. These findings suggest that 15-LOX may have dual and context-dependent roles in vascular tone regulation.

Since ECs and VSMCs express BLT1, BLT2, CysLT1, and CysLT2 receptors, 5-LOX-derived metabolites are closely linked to vascular function [28]. Both LTB4 and CysLTs have been reported to promote VSMCs proliferation and migration and induce vasoconstriction, key pathological features of vascular remodeling in hypertension [9]. One study found that 5-LOX knockout mice attenuated the decreased expression of phenotypic markers in VSMCs following treatment with N(G)-nitro-L-arginine methyl ester (L-NAME), an inhibitor of NO synthase. Conversely, treatment with LTB4 and CysLTs significantly decreased the marker expression levels [110]. Additionally, Selective 5-LOX inhibitors have been effective in alleviating acute microvascular injury induced by NO inhibition in a rat model of endotoxin-induced sepsis [111]. Further studies have shown that L-NAME activates the 5-LOX–LT pathway in human mast cells by inhibiting NO synthase [112]. These findings suggest that 5-LOX and its metabolites (LTB4 and CysLTs) contribute to L-NAME-induced vascular remodeling and hypertension by regulating endothelial inflammation and promoting VSMC phenotypic modulation.

#### Lipoxygenases and Angiogenesis

15-LOX and its metabolites have been implicated in both pathological and tumor angiogenesis [113, 114]. One study demonstrated that hindlimb ischemia-induced angiogenesis, which relies on the 15-LOX-generated metabolite 15-HETE, was more severely impaired in 15-LOX-knockout mice compared to wild-type mice [115]. Additionally, 15-LOX overexpression in human prostate cancer cells has been shown to increase VEGF secretion and promotes angiogenesis [116]. However, some studies suggest that 15-LOX may also inhibit angiogenesis in certain contexts. For instance, the injection of 15-LOX into rabbit skeletal muscle suppressed VEGF-induced angiogenesis, possibly by reducing nitric oxide bioavailability and VEGF receptor 2 expression [117]. Similarly, Viita et al. reported that intravitreal adenoviral 15-LOX gene transfer inhibited VEGF-induced neovascularization in rabbit eyes [118]. Given these conflicting findings, further research is needed to clarify the precise mechanisms by which 15-LOX regulates angiogenesis under different physiological and pathological conditions.

## Conclusions and Future Perspectives

Inflammation plays a critical role in the development and progression of cardiovascular diseases, including atherosclerosis and hypertension. Lipoxygenases (LOXs) are key regulators of both the initiation and resolution of acute inflammation, as they generate pro-inflammatory and pro-resolving lipid mediators through the oxidation of FAs. The metabolites produced by LOXs are substrate-specific and exert distinct effects depending on the cardiovascular condition. For instance, LOX-mediated metabolism of n-6 FAs predominantly generates pro-inflammatory eicosanoids such as LTA4 and LTB4, whereas n-3 FAs-derived metabolites generally exhibit anti-inflammatory properties. Although LOXs and their metabolites are known to be involved in the inflammatory response, their precise role in the pathogenesis of cardiovascular diseases remains inconclusive. Further research utilizing cell- or tissue-specific models, as well as conditional knockout mouse models, may help clarify the specific functions of these enzymes in different cardiovascular conditions. Additionally, targeting key regulatory points in the n-6 and n-3 FA metabolic pathways presents a promising therapeutic strategy for managing cardiovascular and inflammatory diseases.

## Data Availability

Data sharing not applicable to this article as no datasets were generated or analysed during the current study.

## Abbreviations

AA: Arachidonic acid
Ang II: Angiotensin II
cPLA_2_: Cytoplasmic phospholipase A_2_
CYP450: Cytochrome P450
ECs: Endothelial cells
EET: Epoxyeicosatrienoic acid
FLAP: 5-LOX activating protein
HETEs: Hydroxyeicosatetraenoic acids
HODE: Hydroxyoctadecadienoic acids
HPETEs: Hydroperoxyeicosatetraenoic acid
I/R: Ischemia–reperfusion
LA: Linoleic acid
LOX: Lipoxygenase
LRP: LDL receptor-related protein
LTA4: Leukotriene A4
LTB4: Leukotriene B4
LTC4: Leukotriene C4
LTD4: Leukotriene D4
LTE4: Leukotriene E4
LTs: Leukotrienes
MCP-1: Monocyte chemotactic protein-1
oxLDL: Oxidized low-density lipoproteins
PGs: Prostaglandins
PMNL: Polymorphonuclear leukocytes
PUFAs: Polyunsaturated fatty acids
RvD: D-series resolvins
RvE: E-series resolvins
SPMs: Specialized pro-resolving mediators
Th2: T-helper 2 cells
VSMCs: Vascular smooth muscle cells

## Declarations

### Additional information

Associate Editor Junjie Xiao oversaw the review of this article.
